# Continuous versus intermittent short-acting β2-agonists nebulization as first-line therapy in hospitalized children with severe asthma exacerbation: a propensity score matching analysis

**DOI:** 10.1186/s40733-020-00059-5

**Published:** 2020-07-02

**Authors:** Prapasri Kulalert, Phichayut Phinyo, Jayanton Patumanond, Chutima Smathakanee, Wantida Chuenjit, Sira Nanthapisal

**Affiliations:** 1 Department of Clinical Epidemiology, Faculty of Medicine, Thammasat University, Pathum Thani, Thailand; 2 Department of Family Medicine, Faculty of Medicine, Chiang Mai University, Chiang Mai, Thailand; 3 Center for Clinical Epidemiology and Clinical Statistics, Faculty of Medicine, Chiang Mai University, Chiang Mai, Thailand; 4 Department of Pediatrics, Hat Yai Hospital, Songkhla, Thailand; 5 Department of Pediatrics, Faculty of Medicine, Thammasat University, Pathum Thani, Thailand

**Keywords:** Status Asthmaticus, Pediatrics, Hospitalization, Nebulization, Bronchodilators, Propensity scores

## Abstract

**Background:**

Short-acting β2-agonist (SABA) nebulization is commonly prescribed for children hospitalized with severe asthma exacerbation. Either intermittent or continuous delivery has been considered safe and efficient. The comparative efficacy of these two modalities is inconclusive. We aimed to compare these two modalities as the first-line treatments.

**Methods:**

An efficacy research with a retrospective cohort study design was conducted. Hospital records of children with severe asthma exacerbation admitted to Hat Yai Hospital between 2015 and 2017 were retrospectively collected. Children initially treated with continuous salbutamol 10 mg per hour or intermittent salbutamol 2.5 mg per dose over 1–4 h nebulization were matched one-to-one using the propensity score. Competing risk and risk difference regression was applied to evaluate the proportion of children who succeeded and failed the initial treatment. Restricted mean survival time regression was used to compare the length of stay (LOS) between the two groups.

**Results:**

One-hundred and eighty-nine children were included. Of these children, 112 were matched for analysis (56 with continuous and 56 with intermittent nebulization). Children with continuous nebulization experienced a higher proportion of success in nebulization treatment (adjusted difference: 39.5, 95% CI 22.7, 56.3, *p* < 0.001), with a faster rate of success (adjusted SHR: 2.70, 95% CI 1.73, 4.22, *p* < 0.001). There was a tendency that LOS was also shorter (adjusted mean difference − 9.9 h, 95% CI -24.2, 4.4, *p* = 0.176).

**Conclusion:**

Continuous SABA nebulization was more efficient than intermittent nebulization in the treatment of children with severe asthma exacerbation.

## Introduction

Asthma exacerbation is one of the most common causes of hospitalization among children. It was observed that severe asthma exacerbation is increasing in children with asthma [1–3]. Intermittent nebulization with short-acting β_2_-agonist (SABA), salbutamol 0.15–0.3 mg per kg, every one to 4 hours is the current first-line recommendation for hospitalized children with asthma exacerbation [4, 5]. However, children with severe asthma exacerbation may have suboptimal responses to first-line treatment and eventually require an escalation to more aggressive therapy (eg, continuous nebulization, intravenous salbutamol, or intravenous magnesium sulfate). Admission to the pediatric intensive care unit (PICU) is crucial for delivering and monitoring for side effects of these therapies [2, 3, 6]. When progression to life-threatening respiratory failure occurs, endotracheal intubation and mechanical ventilation are needed. The results are asthma complications, prolonged hospital stays, and increased expenditures [3, 7, 8]. Prolonged PICU admission is also a very stressful experience and can be associated with post-traumatic stress disorder for both children and their parents [9, 10].

Continuous nebulization with SABA, salbutamol 10–25 mg per hour or 0.5–1 mg per kg per hour, has been recommended for children with asthma exacerbation who did not show adequate response to the first-line intermittent nebulization [11–13]. It was reported that some children with severe asthma exacerbation usually ended up with continuous nebulization therapy [2]. In the past, the continuous mode was limited to only life-threatening cases and must be administered in the PICU due to the general perception of its potentially serious side effects when used in children (eg, diastolic hypotension, or arrhythmia). However, one study in 2014 reported various aspects of clinical improvements in severe asthma symptoms, lesser incidences of intensive care unit transfer, fewer patients requiring respiratory support, and sufficient safety when albuterol was administered continuously as the first-line treatments among children in non-PICU settings [14].

A proper asthma treatment strategy should aim to rapidly relieve the symptoms and reduce the chance of asthma complications. Early aggressive therapy may be an alternative solution. Since 2015, our hospital has developed a treatment protocol that allows the initiation of either continuous or intermittent nebulization as the first-line therapy in children with severe asthma exacerbation, depending on physicians’ preferences. To the best of our knowledge, no study has yet compared the clinical efficacy between continuous and intermittent SABA nebulization as the first-line treatment in children with severe asthma exacerbation. We hypothesized that early administration of continuous SABA nebulization might increase the rate of successful treatment and could shorten the time to the resolution of asthmatic symptoms. The objective of our study was to investigate the clinical efficacy of continuous SABA nebulization as the first-line treatment instead of intermittent SABA nebulization in children with severe asthma exacerbation.

## Methods

### Study design and patient cohort

This therapeutic study was conducted based on a retrospective cohort of children with asthma exacerbation who were admitted at Hat Yai Hospital, a tertiary care hospital in Songkhla (province) between January 1, 2015 and December 31, 2017. Asthma diagnosis was identified by the International Classification of Diseases, Tenth Revision, and Clinical Modification codes (ICD-10) with the asthma discharge diagnosis codes J45 to 46. The requirement for informed consent was waived as the data were retrospectively collected and were anonymous. The study was approved by the Institutional Review Board of the Hat Yai Hospital (protocol number 33/2561) and the Faculty of Medicine, Thammasat University (MTU-EC-ES-0-061/61).

### Study participants

All admission records during the study period were screened for eligibility. Only children who fulfilled all of the following criteria were included for analysis: 1) age between 1 and 15 years, 2) prior diagnosis of asthma or had at least two episodes of bronchodilator-responsive wheezing if no asthma diagnosis was documented, 3) having features of severe asthma exacerbation at initial admission, according to British Guidelines on the Management of Asthma (Table 1) [15], and 4) had been administered with continuous salbutamol nebulization 10 mg/h or intermittent salbutamol nebulization 2.5 mg per dose as first-line therapy. For accurate asthma diagnosis, children aged less than 12 months were excluded. Children were excluded if they were prescribed with other first-line therapy, such as adrenaline or 3% NaCl nebulization, had any comorbidity eg, chronic cardiopulmonary diseases. We also excluded those who were referred from other hospitals, those who were referred to another hospital before complete resolution of asthma exacerbation, and those whose data on prognostic factors, emergency room (ER) treatment, clinical asthma severity on initial admission, and co-medications during admission were missing.

**Table 1:** Diagnosis of severe asthma exacerbation according to the British Guideline on the Management of Asthma for children in hospital

Age (years)	Severe asthma
Age 1–5	SpO_2_ < 92%
	Too breathless to talk or eat
	HR > 140/min
	RR > 40/min
	Use of accessory neck muscles
	Without features of life-threatening asthma
Age > 5	SpO_2_ < 92%
	PEF 33–50% best or predicted
	Too breathless to talk or eat
	HR > 125/min
	RR > 30/min
	Use of accessory neck muscles
	Without features of life-threatening asthma

Abbreviations: *HR* Heart rate; *PEF* Peak expiratory flow; *RR* Respiratory rate; *SpO*_*2*_ Room air oxygen saturation

### Treatment groups

Included children were coded as two groups according to their initial treatment at the pediatric ward. The salbutamol solution was prepared by diluting 2 mL of Ventolin Respirator solution® (5 mg/mL) in 28 mL of normal saline solution for both groups. For the first group, continuous nebulization of salbutamol was delivered by High Output Extended Aerosol Respiratory Therapy (HEART) via a face mask with an oxygen flow rate of 10 l per minute at a concentration of 10 mg per hour [16]. The second group was treated with intermittent nebulization of salbutamol. The dose of salbutamol was 1.5 mg for children weighed < 10 kg and 2.5 mg for children weighed ≥10 kg. Salbutamol was delivered interval via a face mask with an oxygen flow rate of 6–8 l per minute at 1 to 4 h intervals [4, 5].

Other than the first-line treatments, all children were provided with as standard asthma treatment: systemic corticosteroids given at recommended doses with supplemental oxygen to maintain oxygen saturation (SpO_2_) > 95% [4, 5, 15]. Additional therapy during admission was given as needed **(**eg, ipratropium bromide nebulization or subcutaneous terbutaline injection) depending on physician’s judgment.

Like most hospitals, we employ a stepwise approach for the treatment of children with severe asthma exacerbation. The usual steps in escalating therapy in children with asthma exacerbation are as follows**:** 1) SABA delivered by intermittent nebulization, 2) continuous nebulization of SABA, 3) continuous intravenous terbutaline as adjunctive treatment, 4) If the children develop respiratory failure, endotracheal intubation and mechanical ventilator are used. Our step-down protocol is to provide each child with 10 mg per hour of continuously nebulized salbutamol until the children’s respiratory status improves. After that, the treatment would be changed to intermittent nebulization of salbutamol, 2.5 mg per dose, with sequentially decreasing of frequency (eg, every 1 h, 2 h, 3 h, and 4 h).

### Study endpoints

The primary endpoint was the proportion of children who succeeded in the initial first-line treatment. Failure of initial treatment was defined as the escalation of treatment to more aggressive treatment. For intermittent nebulization therapy, treatment failure were defined as the requirement of at least one of the following treatments: continuous nebulization, intravenous terbutaline, continuous positive airway pressure, bi-level positive airway pressure, or mechanical ventilation. Children in the continuous nebulization group were considered to have failed if they subsequently required at least one of the following: intravenous terbutaline, ventilator support with continuous positive airway pressure, bi-level positive airway pressure, or mechanical ventilation. The secondary endpoint was the length of hospital stay (LOS).

### Pre-specified confounders

#### Confounding by indication

The characteristics that may affect physicians’ treatment selection were the age of the children and the clinical severity of asthma exacerbation (eg, SpO_2_, respiratory rate, and pulse rate) [13]. Therefore, we defined age, respiratory rate, pulse rate, and oxygen saturation as confounding by indication and used them to derive the propensity scores.

#### Prognostic factors

Our prognostic factors were those that predisposed children toward the risk of asthma-related death or near-fatal asthma, PICU admission, or mechanical ventilation. These include 1) history of endotracheal intubation and mechanical ventilation for asthma exacerbation [17, 18], 2) ≥1 asthma exacerbation (either hospitalization or ER visit for asthma) in the past 12 months [17], 3) not currently using controller medication [11, 17], 4) exacerbation triggered by pneumonia [14, 19], 5) subcutaneous injections of terbutaline at ER [14], or 6) obesity [20]. As not all children had the data on height, obesity was defined as a percentile of weight for age exceeding 90 [21].

#### Pretreatment confounders and confounders by co-medications

Medications for the treatment of asthma exacerbation during an ER and hospital admission may affect the children’s clinical outcomes. Nebulized salbutamol < 3 doses at ER and time to the first dose of systemic corticosteroid were defined as pre-treatment confounders [17]. Apart from the first-line SABA treatment during admission, co-medications (eg, subcutaneous injection of terbutaline, or ipratropium bromide nebulization) might be prescribed to some children depending on physicians’ judgment and could potentially affect our study endpoints [5, 11, 12]. Two medications were defined as confounders by co-medications. Therefore, in this study, both pre-treatment confounders and confounders by co-medications were considered during statistical analysis.

### Data collection

Patient characteristics, including age, gender, weight, history of asthma diagnosis, history of using asthma controller medications, number of ER visits in the past 12 months, number of hospitalizations in the past 12 months, and history of intubation for asthma exacerbation were collected. Details on all medications for the treatment of asthma exacerbation at ER and during hospital admission were also collected. We recorded the time of initiation nebulization treatment, the time of admission to the ward or PICU, as well as the time of treatment cessation, and the time that physicians ordered discharge. Adverse side effects during nebulization (eg, nausea, vomiting, tremors, or diastolic hypotension) were observed and documented.

### Statistical analysis

Data were analyzed using Stata version 16 (StataCorp, Lakeway, Texas, USA). For all statistical analyses, a two-sided *p*-value less than 0.05 was considered as significant. Descriptive statistics were calculated for all clinical characteristics and relevant variables; frequencies were calculated for categorical variables and presented as percentages. Mean and standard deviation or median and interquartile range were calculated for continuous variables, as appropriate. We used standardized difference (STD) to measure the magnitude of differences in clinical characteristics, prognostic factors, and potential confounders between treatment groups, where an absolute STD of less than 10% was considered as no significant difference between groups [22].

As this was non-randomized therapeutic research, selection bias and imbalanced of prognostic determinants were likely to occur. We, therefore, performed a propensity score matching between the two groups before the estimation of the treatment effects. These techniques are regarded as acceptable for observational (non-randomized) studies [22–26]. We first calculated the propensity score to estimate the probability for each child to be initially treated with continuous nebulization by logistic regression. Our propensity score model included age, respiratory rate, pulse rate, and oxygen saturation on initial admission. The derived propensity scores were split into different blocks with a similar probability of being treated with continuous nebulization. Within each block, we matched the children who were treated with continuous nebulization to the children who were treated with intermittent nebulization with a one-to-one ratio. We then assessed the balance of baseline characteristics, prognostic factors, and potential confounders between the two groups after matching with STD.

Risk difference regression was applied to compare both the proportion of succeeding the first-line treatment and the proportion of failing the first-line treatment between treatment groups. As a failure of the first-line treatment could preclude the endpoint of interest and alter the probability of succeeding the first-line treatment, treatment failure is thus a competing risk in this clinical context. We used competing risk regression to estimate the cumulative incidence function for treatment success and treatment failure for continuous nebulization over intermittent nebulization. The results of competing risk analysis were presented as subdistribution hazard ratio (SHR), according to Fine and Gray [27]. In addition, the restricted mean survival time difference (RMST) was used to compare the differences in the LOS between both groups. The RMST analysis was based on flexible parametric modeling of log cumulative hazards with 3 d.f. for baseline hazard distribution and 1 d.f. for time-treatment interaction, as suggested by Royston and Parmar [28]. To entirely eliminate residual confounding, we adjusted all regression models with all remaining prognostic factors, pre-treatment confounders, and confounders by co-medications.

## Results

**Fig. 1:**
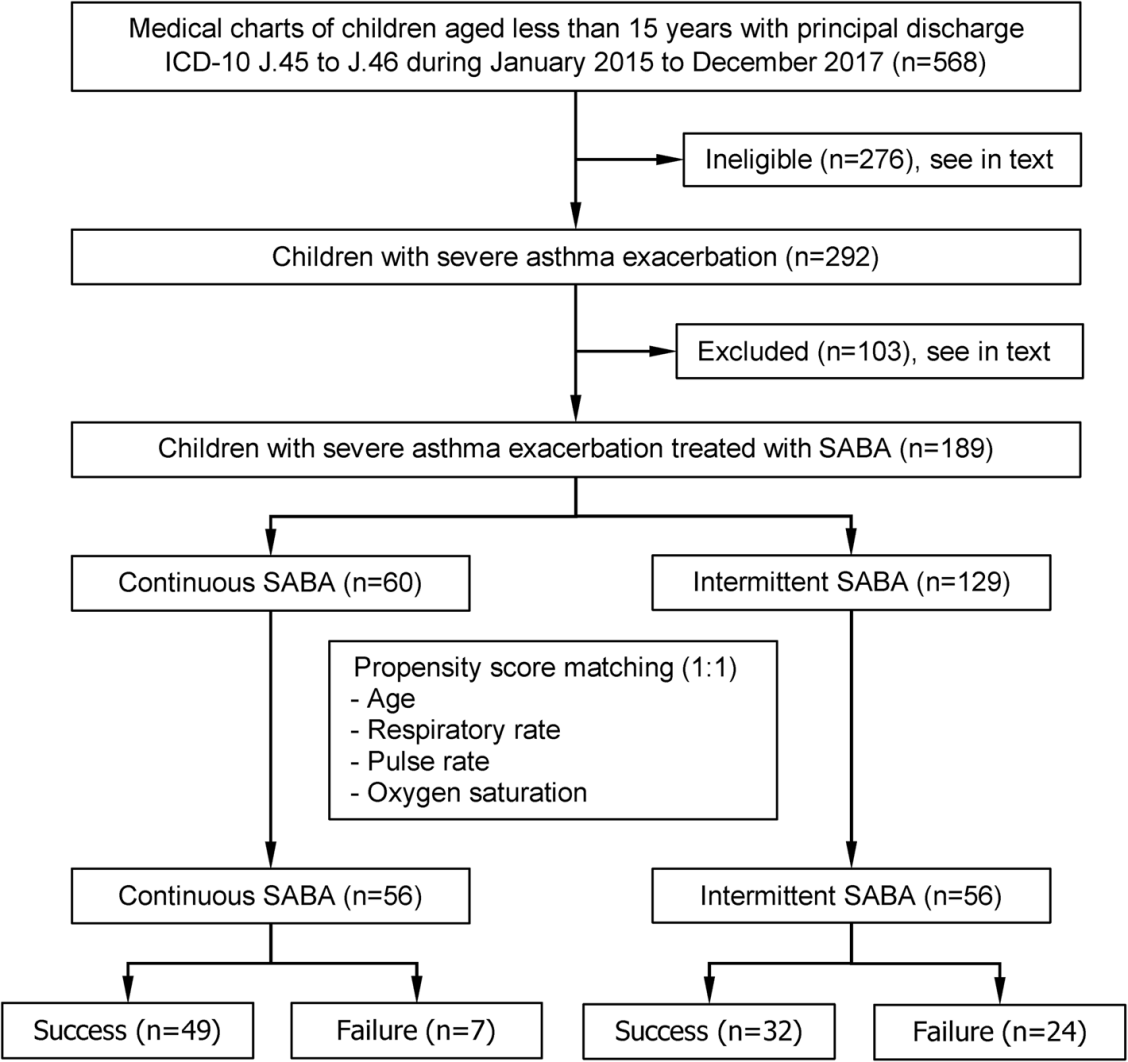
Study flow diagram of the patient cohort

A total of 568 medical charts of hospitalized children from January 2015 to December 2017 were screened. Of these, 276 did not fulfill the inclusion criteria: 154 had moderate asthma exacerbation, 41 were not diagnosed with asthma and had less than 2 episodes of bronchodilator-responsive wheezing, 34 were less than 12 months old, 22 had life-threatening asthma exacerbation, 21 were not diagnosed with asthma on admission (eg, bronchiolitis or anaphylaxis), and 4 were treated with different treatment protocols of SABA. Two-hundred and ninety-two children with severe asthma exacerbation met the inclusion criteria. One-hundred and three children were excluded due to following reasons: 48 had missing data on emergency treatments, 21 had cardiopulmonary diseases, 15 had first-line therapy with other medications, 12 were referred to other hospitals before complete resolution of exacerbation, 4 had missing data on clinical asthma severity on initial admission, and 3 were referred from other hospitals (Fig. 1).

One hundred and eighty-nine children were included in this study, 60 were initially treated with continuous nebulization and 129 with intermittent nebulization. The comparison of patient clinical characteristics, prognostic factors, potential confounders, and propensity scores was shown in Table 2. Age, respiratory rate, pulse rate, and oxygen saturation on admission differed greatly between the two treatment groups with a standardized difference of more than 10% in all variables. After one-to-one propensity score matching, we had 112 children divided evenly between initial continuous and intermittent nebulization (56:56). The propensity score model was shown in Table 3.

**Table 2:** Clinical characteristics, prognostic factors, and potential confounders of the study patients

Characteristics	Original cohort					Propensity-matched cohort				
	Continuous nebulization (*n* = 60)		Intermittent nebulization (*n* = 129)		STD	Continuous nebulization (*n* = 56)		Intermittent nebulization (*n* = 56)		STD
	n	(%)	n	(%)	(%)	n	(%)	n	(%)	(%)
**Clinical characteristics**										
Age (years), median (IQR)	5.6	(3.3,8.9)	4.2	(2.6,7.6)	−34.1	5.6	(3.3,8.7)	5.6	(3.2,8.6)	−0.45
Male gender	37	(61.7)	84	(65.1)	7.12	33	(58.9)	33	(58.9)	0
Weight for age (%), median (IQR)	23.6	(6.7,73.1)	28.4	(3.9,75.5)	2.87	27	(10.5,74.7)	23	(2.9,80.7)	−2.87
**Prognostic factors**										
Obesity	10	(16.7)	16	(12.4)	−12.04	10	(17.9)	9	(16.1)	−4.72
Not currently using controller medication	22	(36.7)	59	(45.7)	18.39	21	(37.5)	27	(48.2)	21.58
≥ 1 exacerbation in the past 12 months	45	(75.0)	97	(75.2)	0.45	42	(75.0)	41	(73.2)	−4.04
History of endotracheal intubation	7	(11.7)	12	(9.3)	−7.67	7	(12.5)	4	(7.1)	−17.91
Exacerbation triggered by pneumonia	18	(30.0)	50	(38.8)	18.41	17	(30.4)	19	(33.9)	7.58
**Treatment at emergency room**										
Nebulized salbutamol < 3 doses	8	(13.3)	25	(19.4)	16.31	8	(14.3)	12	(21.4)	18.56
Terbutaline subcutaneous injection	10	(16.7)	8	(6.2)	−33.11	9	(16.1)	4	(7.1)	−27.90
Time to first dose of systemic corticosteroids (min), median (IQR)	53	(22,155)	80	(29,175)	5.09	53	(23.5151)	70	(25,149.5)	−11.40
**Clinical data on initial admission**										
Respiratory rate (per min), mean ± SD	40.8	±8.7	39.8	±7.5	−12.50	40.7	±8.1	40.5	±8.0	−1.78
Pulse rate (per min), mean ± SD	143.5	±16.9	147.2	±16.7	22.04	144.3	±16.5	143.6	±16.4	−4.12
Oxygen saturation (%), mean ± SD	93.9	3.2	93.5	±3.4	−11.41	93.8	±3.3	93.6	±3.6	−6.23
**Co-medications during admission**										
Ipratropium bromide nebulization	25	(41.7)	37	(28.7)	−27.27	23	(41.1)	17	(30.4)	−22.30
Terbutaline subcutaneous injection	31	(51.7)	15	(11.6)	−94.70	28	(50.0)	7	(12.5)	−87.67
Propensity score, mean ± SD	0.37	±0.14	0.29	±0.12	−62.56	0.35	±0.13	0.35	±0.13	−0.62

Abbreviation: *IQR* Interquartile range; *SD* Standard deviation; *STD* Standardized difference

**Table 3:** Derivation of propensity scores via multivariable logistic regression model

Equation parameters	Coefficient	Standard error	95% confidence interval	*P*-value
Age function 1^a^	−2.96	1.63	−6.15, 0.23	0.069
Age function 2^a^	0.01	0.01	−0.01, 0.01	0.147
Respiratory rate function 1^a^	35,024.04	17,178.24	1355.31, 68,692.77	0.041
Respiratory rate function 2^a^	−11,823.00	5542.34	−22,685.78, − 960.22	0.033
Pulse rate function 1^a^	− 284,024.70	707,345.10	−1,670,396.00, 1,102,346.00	0.688
Pulse rate function 2^a^	66,655.37	160,989.00	− 248,877.30, 382,188.10	0.679
Oxygen saturation function 1^a^	− 815,886.90	2,937,831.00	−6,573,930.00, 4,942,156.00	0.781
Oxygen saturation function 2^a^	197,629.00	731,112.40	−1,235,325.00, 1,630,583.00	0.787
Constant (intercept)	−6.93	43.00	− 91.20, 77.35	0.872

^a^Optimal second-degree fractional polynomial terms for age, respiratory rate, pulse rate and oxygen saturation parameters

Table 2 also showed the clinical characteristics, prognostic factors, and potential confounders of the propensity score matched cohort. After matching, age, respiratory rate, pulse rate, and SpO_2_ were acceptably similar with a standardized difference less than 10%. For prognostic factors, there were no notable discrepancies in the proportions of obese children, children with ≥1 asthma exacerbation episode in the past 12 months, and children whose exacerbation were triggered by pneumonia. However, the proportion of children having a history of intubation for asthma and not currently using controller medication were significantly different (STD − 17.91 and STD 21.58, respectively).

Children in the continuous nebulization group had a lower proportion of nebulized salbutamol < 3 doses at ER (14.3% vs. 21.4%, STD = 18.56%), a higher proportion of subcutaneous terbutaline injection (16.1% vs. 7.1%, STD = -27.90%), and lower time to the first dose of systemic corticosteroids (median time 53 vs. 70 min, STD = -11.40%). During admission, there was a significantly higher proportion of ipratropium bromide nebulization (41.1% vs. 30.4%, STD − 22.3%) and subcutaneous injection of terbutaline (50.0% vs. 12.5%, STD − 87.67%) in the continuous nebulization group compared to the intermittent nebulization group.

**Fig. 2:**
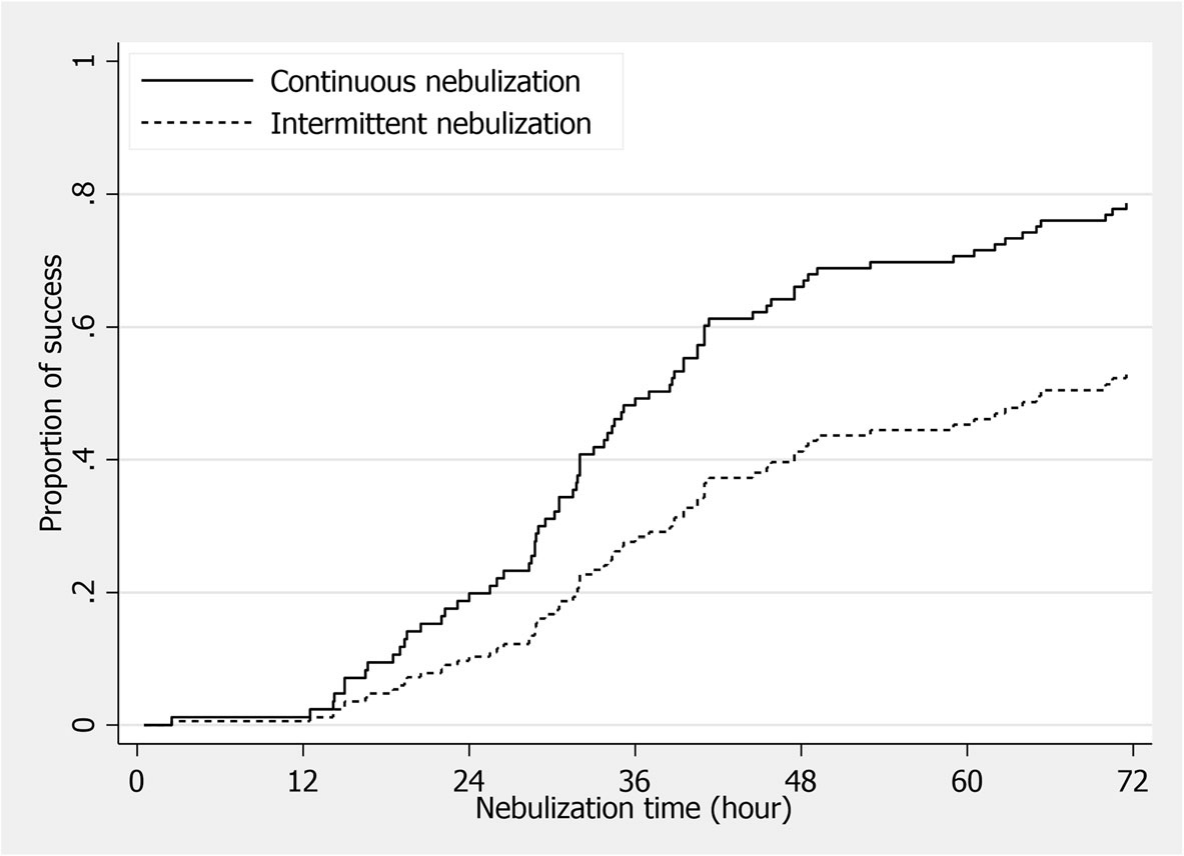
Competing risk estimates for cumulative incidence of success in nebulization

**Fig. 3:**
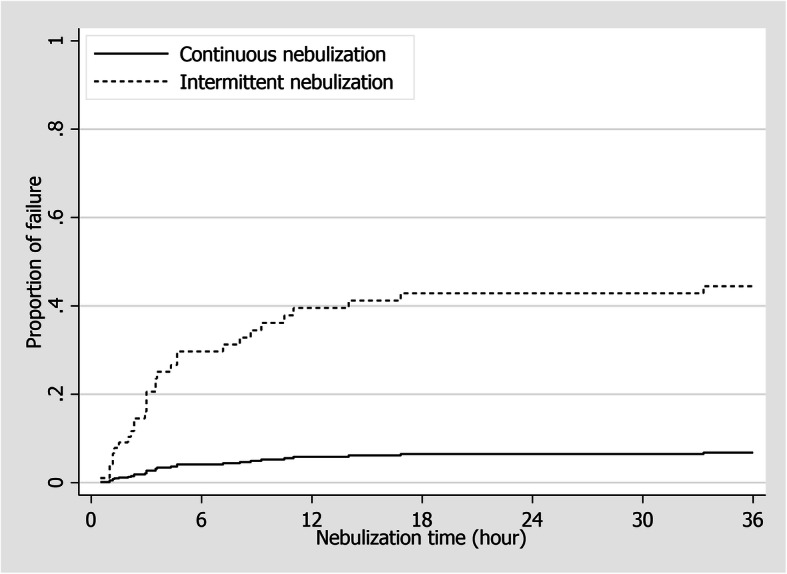
Competing risk estimates for cumulative incidence of failure in nebulization

Forty-nine children in the continuous nebulization group succeeded in the initial treatment, and only seven required escalating therapy with intravenous terbutaline injection. In the intermittent nebulization group, 32 children succeeded the initial treatment, 21 children were escalated to continuous nebulization with SABA, two were administered with intravenous terbutaline, and one child was mechanically ventilated. The results of all pre-specified clinical endpoints were presented in Table 4. Children in the continuous nebulization group had a higher proportion of success compared to children in the intermittent nebulization group (adjusted risk difference 39.5, 95% CI 22.7 to 56.3, *p* < 0.001). The rate of successful first-line nebulization treatment was also higher in the continuous nebulization group (adjusted SHR: 2.70, 95% CI 1.72 to 4.22, *p* < 0.001) (Fig. 2). The risk of failing initial treatment was also found to be lower in children treated with continuous nebulization (adjusted risk difference − 39.5, 95% CI − 56.3 to − 22.7, *p* < 0.001). The cumulative incidence of failing initial treatment was significantly lower in the continuous nebulization group (adjusted SHR: 0.12, 95% CI 0.05 to 0.31, p < 0.001) (Fig. 3).

**Table 4:** Pre-specified study clinical endpoints

Clinical endpoints	Continuous nebulization (*n* = 56)		Intermittent nebulization (*n* = 56)		Treatment effect (continuous vs. intermittent nebulization)						
	n	%	n	%	Clinical parameters	Unadjusted analysis			Adjusted analysis^a^		
						Effect	95%CI	*P*-value	Effect	95%CI	*P*-value
Success	49	87.5	32	57.1	Risk difference (%)	30.4	14.6, 46.1	< 0.001	39.5	22.7, 56.3	< 0.001
					SHR	2.03	1.30, 3.17	0.002	2.70	1.72, 4.22	< 0.001
Failure	7	12.5	24	42.9	Risk difference (%)	−30.4	−46.1, −14.6	< 0.001	−39.5	−56.3, −22.7	< 0.001
					SHR	0.25	0.11, 0.58	0.001	0.12	0.05, 0.31	< 0.001
LOS (hour), mean ± SD	52.8	±28.2	59.1	±66.4	RMST difference (mean)	−2.92	−14.54, 8.70	0.623	−9.88	−24.18, 4.42	0.176

Abbreviation: *LOS* Length of stay; *RMST* Restricted mean survival time; *SHR* Sub-distributional hazard ratio (under competing risk time-to-event analysis).^a^multivariable analysis adjusted for potential confounders (prognostic factors, treatment at emergency room, and co-medications during admission)

The mean LOS was 52.8 ± 28.2 h in the continuous nebulization group and 59.1 ± 66.4 h in the intermittent nebulization group. After controlling for potential confounders, there was a tendency that the administration of continuous nebulization as a first-line treatment instead of intermittent nebulization could reduce the children’s LOS by almost 10 h (adjusted RMST difference − 9.88 h, 95% CI − 24.18 to 4.42, *p* = 0.176). For treatment-related adverse events, only one child who was treated with continuous nebulization reported nausea and vomiting. These symptoms disappeared after treatment cessation.

## Discussion

According to our results, children with severe asthma exacerbation who were initially treated with continuous nebulization showed a significantly higher rate of treatment success and a lower chance of treatment failure and requirement of escalation to a more aggressive therapy compared to children who were treated with intermittent nebulization. Moreover, none of the children in the continuous nebulization group was intubated, whereas one of the children in the intermittent nebulization group was. Continuous nebulization as the first-line therapy also showed a tendency to reduce the LOS.

In terms of treatment efficacy, previous studies had reported and concluded the superiority of continuous nebulization with SABA over intermittent nebulization [29–32]. However, the patient’s clinical characteristics, outcome measurements, and health care setting in these studies differed from ours. Two meta-analyses [29, 31] and one randomized trial [30] selectively included only children who presented to the ER. It was concluded from these studies that children who were treated with continuous nebulization had an improvement in asthma score at 2 h after treatment and had shorter hospitalization. One study showed that continuous nebulization with albuterol significantly improved asthma outcomes in children with asthma exacerbation who presented with impending respiratory failure in a PICU setting [32]. The children in this study carried higher clinical severity of asthma exacerbation than the children within our study. Thus, the generalization of results from these studies to our clinical circumstance was not plausible. Another study evaluated the efficacy of continuous albuterol nebulization as the first-line treatment in children who were hospitalized with severe asthma exacerbation [14]. Only 5 % of children with severe asthma exacerbation had clinical deterioration and required enhanced respiratory support (ie, using continuous positive airway pressure, or bi-level positive airway pressure) or transferred to PICU. No children were intubated. However, the study did not directly compare the efficacy of continuous nebulization among children with severe asthma exacerbation against that of intermittent nebulization.

Our study might be the first to directly compare the efficacy of continuous and intermittent SABA delivery as first-line treatment in children with severe asthma exacerbation admitted to general pediatric wards. Children with severe asthma exacerbation accounts for a large proportion of hospitalized children, and the trend seemed to be increasing [1–3]. These children usually have a suboptimal response to intermittent SABA nebulization and may require aggressive stepwise therapy, or may develop respiratory failure and end up with asthma-related complications and prolonged hospital stay. Our study showed a lower proportion of children who required escalation of treatment and a higher rate of successful first-line treatment in the continuous nebulization group than in the intermittent nebulization group. In addition, no children in the continuous nebulization group developed respiratory failure and required endotracheal intubation. The reduction in the number of intubated cases would lead to fewer asthma complications, decreased health care costs, and shortened the LOS [3, 7, 8].

Children in the continuous nebulization group had a shorter LOS of approximately 10 h, as compared to those in the intermittent nebulization group; however, the finding did not yield a statistical significance. This might be the consequence of limits in statistical power due to the small study size. However, considering the large volume of pediatric asthma hospitalizations, even a small reduction in the LOS can decrease a large economic burden, eg, a reduction of 0.5 days in median LOS could save $160 million [33, 34]. However, as the burden of hospitalization due to asthma exacerbation varies by countries, and continuous therapy is more expensive than intermittent one. Future studies should aim to assess the cost-effectiveness of such approach with a larger population-based study.

Due to concerns regarding serious adverse effects of continuous nebulization with SABA (ie, diastolic hypotension, or arrhythmia), the treatment was strictly limited to children with a very severe or life-threatening asthma exacerbation who were also needed to be admitted to PICU before the administration of continuous therapy. These serious side effects are known to be dose-dependent and commonly occur when given at a high dose 25 mg per hour, 75 mg per hour and 150 mg per hour [35–37]. In this study, a lower dose, 10 mg per hour, was used, which was relatively safe compared to the higher dose. There was only one child who developed nausea and vomiting. Previous studies also supported the clinical safety of the dosage used in this study [14, 37].

To estimate causal effect between applying continuous nebulization as the first-line therapy and the improvements in asthma endpoints from observational data, we used the propensity score method to minimize the presence of selection bias and balance the distribution of measured covariates that give rise to confounding by indication or confounding by contraindication [22]. In this study, propensity matching was used for the derivation of a comparable cohort of continuous and intermittent nebulization therapy [22, 38]. The score was based on four pre-specified characteristics that influence the selection of treatments, either continuous or intermittent nebulization, by clinicians, which were age, respiratory rate, pulse rate, and oxygen saturation upon admission. After matching, the existing differences between groups became smaller; however, the remaining prognostic factors and potential confounders were imbalance with STD > 10%. Concluding the treatment effect in the presence of a significant imbalance of covariates could compromise the result. Therefore, we performed double adjustment by multivariable adjustment of remaining prognostic factors and potential confounders with STD > 10% [39]. This approach could substantially eliminate residual confounding bias, increase statistical power [40], and improve the validity of causal estimate [41].

There were some limitations to be addressed. First, we could not perform peak expiratory flow rate (PEFR) measurements to assess the lung function and treatment response to all children who were older than 5 years old, as we did not have enough peak flow meter in the past. Besides, approximately half of our patients aged less than 5 years old. In this study, pulse oximetry, another objective measurement suggested by the Global Initiative for Asthma (GINA) guidelines [17], was used to assess the children before and after treatment. Second, the data used for analysis were retrospectively collected. All nebulization techniques were done according to our routine practice without standard operating procedures as in clinical trials. However, our health care team generally follows local guidelines on how to deliver and monitor the side effects of all medications. Thus, although the data might not be in a well-controlled environment, it did reflect the real-world effectiveness of both treatments in our setting. Third, the study size after propensity score matching was small and might not be adequate to power the multivariable regression analysis. However, as the treatment effect was substantial, the effect estimates were acceptably precise. Fourth, even after propensity score matching and multivariable adjustment of all measured confounders, residual confounding from unmeasured covariates may remain and could confound the treatment effects. A future randomized clinical trial to evaluate the efficacy of continuous nebulization therapy as the first-line treatment in hospitalized children with severe asthma exacerbation should be done to confirm the results of our study.

In conclusion, the administration of continuous salbutamol nebulization as first-line therapy in children with severe asthma exacerbation revealed a higher success rate and decreased in need for escalation of therapy. It should be considered as an alternative first-line treatment for hospitalized children with severe asthma exacerbation.

## Data Availability

The datasets used and/or analyzed during the current study are available from the corresponding author on reasonable request.

## Abbreviations

SABA: Short-acting β_2_-agonist
PICU: Pediatric intensive care unit
ICD: International classification of diseases
ER: Emergency room
HEART: High output extended aerosol respiratory therapy
SpO_2_: Oxygen saturation
STD: Standardized difference
SHR: Subdistribution hazard ratio
RMST: Restricted mean survival time
LOS: Length of hospital stay
PEFR: Peak expiratory flow rate
GINA: Global initiative for asthma
